# From Arylamine *N-*Acetyltransferase to Folate-Dependent Acetyl CoA Hydrolase: Impact of Folic Acid on the Activity of (HUMAN)NAT1 and Its Homologue (MOUSE)NAT2

**DOI:** 10.1371/journal.pone.0096370

**Published:** 2014-05-13

**Authors:** Nicola Laurieri, Julien Dairou, James E. Egleton, Lesley A. Stanley, Angela J. Russell, Jean-Marie Dupret, Edith Sim, Fernando Rodrigues-Lima

**Affiliations:** 1 Department of Pharmacology, University of Oxford, Oxford, United Kingdom; 2 Department of Chemistry, Chemistry Research Laboratory, University of Oxford, Oxford, United Kingdom; 3 Université Paris Diderot, Sorbonne Paris Cité, Unit of Functional and Adaptive Biology, Paris, France; 4 Consultant in Investigative Toxicology, Linlithgow, West Lothian, United Kingdom; 5 Faculty of Science, Engineering and Computing, Kingston University, Kingston on Thames, United Kingdom; University of Hawaii Cancer Center, United States of America

## Abstract

Acetyl Coenzyme A-dependent *N-*, *O-* and *N,O-*acetylation of aromatic amines and hydrazines by arylamine *N-*acetyltransferases is well characterised. Here, we describe experiments demonstrating that human arylamine *N-*acetyltransferase Type 1 and its murine homologue (Type 2) can also catalyse the direct hydrolysis of acetyl Coenzyme A in the presence of folate. This folate-dependent activity is exclusive to these two isoforms; no acetyl Coenzyme A hydrolysis was found when murine arylamine *N-*acetyltransferase Type 1 or recombinant bacterial arylamine *N-*acetyltransferases were incubated with folate. Proton nuclear magnetic resonance spectroscopy allowed chemical modifications occurring during the catalytic reaction to be analysed in real time, revealing that the disappearance of acetyl C*H*_3_ from acetyl Coenzyme A occurred concomitantly with the appearance of a C*H*_3_ peak corresponding to that of free acetate and suggesting that folate is not acetylated during the reaction. We propose that folate is a cofactor for this reaction and suggest it as an endogenous function of this widespread enzyme. Furthermore, *in silico* docking of folate within the active site of human arylamine *N-*acetyltransferase Type 1 suggests that folate may bind at the enzyme’s active site, and facilitate acetyl Coenzyme A hydrolysis. The evidence presented in this paper adds to our growing understanding of the endogenous roles of human arylamine *N-*acetyltransferase Type 1 and its mouse homologue and expands the catalytic repertoire of these enzymes, demonstrating that they are by no means just xenobiotic metabolising enzymes but probably also play an important role in cellular metabolism. These data, together with the characterisation of a naphthoquinone inhibitor of folate-dependent acetyl Coenzyme A hydrolysis by human arylamine *N-*acetyltransferase Type 1/murine arylamine *N-*acetyltransferase Type 2, open up a range of future avenues of exploration, both for elucidating the developmental role of these enzymes and for improving chemotherapeutic approaches to pathological conditions including estrogen receptor-positive breast cancer.

## Introduction

Arylamine *N-*acetyltransferases (NATs) are well characterised xenobiotic-metabolising enzymes which catalyse the acetyl Coenzyme A (AcCoA)-dependent *N-*, *O-* and *N,O-*acetylation of aromatic amines and hydrazines. They have also been shown to play important endogenous roles as well as having potential as novel targets for pharmacological intervention [1], [2].

The human genome contains two adjacent NAT genes and a pseudogene (*NATP*). Both functional genes encode enzymes which catalyse the transfer of an acetyl group from AcCoA to an arylamine resulting in the formation of an amide bond, although their individual substrate specificities differ [2], [3]. One of these enzymes, arylamine *N-*acetyltransferase Type 2 ((HUMAN)NAT2), is responsible for the acetylation of a range of drugs and xenobiotics and came to prominence because of its role in the polymorphic metabolism of the anti-tubercular agent isoniazid. Specific combinations of single nucleotide polymorphisms (SNPs) in the *(HUMAN)NAT2* gene are associated with slow acetylation of a number of therapeutic agents as well as industrial chemicals such as arylamines.

The second functional human NAT gene encodes human arylamine *N-*acetyltransferase Type 1 ((HUMAN)NAT1) and has also been shown to be polymorphic, although the consequences of the individual SNPs at this locus have been called into question because many of them are outside the coding region [4]. The substrate specificity of (HUMAN)NAT1 differs from that of (HUMAN)NAT2 in that its preferred model substrate is the arylamine *para*-aminobenzoic acid (pABA) rather than the arylhydrazine compounds preferred by (HUMAN)NAT2.

Determination of the crystal structures of (HUMAN)NAT1 and (HUMAN)NAT2 has allowed their substrate specificities to be explained. These studies have shown that the catalytic triad of Cys69, His107 and Asp122 initially identified in the prokaryotic NAT of *Salmonella typhimurium* ((SALTY)NAT) [5] has the same orientation in both human NAT enzymes in which Cys is located at position 68 and His at 106 [6]. The architecture of the active sites of (HUMAN)NAT1 and (HUMAN)NAT2 reinforced earlier evidence identifying the importance of residues 125–129 in determining substrate specificity [7] and substantiated the importance of the *C*-terminus in controlling activity, as originally demonstrated by Sinclair and Sim [8]. These observations help to explain why, whilst (HUMAN)NAT1 metabolises a spectrum of substrates which overlaps that of (HUMAN)NAT2, its specificity profile is unique.

While (HUMAN)NAT1 is structurally similar to (HUMAN)NAT2, it is biologically distinct in that it is expressed during embryonic development (as early as the four-cell stage), in placenta during trimester one and throughout pregnancy [9]–[11], and in stem cells. The enzymatic profile of NAT1 expression in different human foetal organs [12] illustrates its widespread tissue distribution, which differs from the much more restricted distribution of (HUMAN)NAT2, and many strands of evidence suggest a link between (HUMAN)NAT1 and folate [13]–[15].

The potential role of (HUMAN)NAT1 in folate clearance in the embryo and placenta is of particular interest because of the fact that folic acid supplementation is effective in the prevention of neural tube defects (NTDs) [16]. Rare loss-of-function mutations in *(HUMAN)NAT1* are associated with reduced risk of spina bifida, and *(HUMAN)NAT1* genotype has therefore been identified as a risk factor for this condition [17]. The chromosomal region marked by *(HUMAN)NAT1* and *(HUMAN)NAT2* has also been shown to influence the incidence of sporadic and teratogen*-*induced orofacial clefting [18]. Moreover, folate supplementation during pregnancy seems to reduce the incidence of oral clefts in newborns [19].

Like humans, mice have two functional *NAT* genes [20]–[22]. Historically, the characterisation of NAT polymorphisms in the mouse preceded the discovery of polymorphism in (HUMAN)NAT1; the polymorphic murine NAT is therefore designated (MOUSE)NAT2 by analogy with (HUMAN)NAT2 [23], despite the fact that it shares the wide tissue distribution, pattern of expression during embryonic development [24] particularly in foetal neural tissues [25] and substrate specificity of (HUMAN)NAT1 [20].

**Figure 1:**
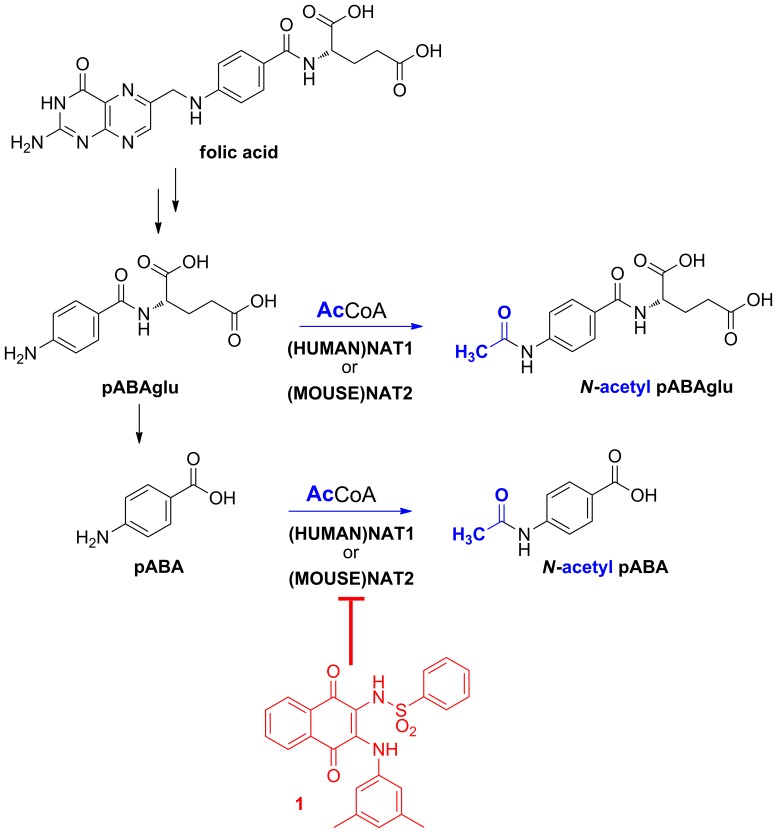
Potential role of (HUMAN)NAT1 and (MOUSE)NAT2 in folate degradation. One endogenous role of (HUMAN)NAT1 and (MOUSE)NAT2 appears to be related to the breakdown of folate through the acetylation of the folate catabolites pABAglu and pABA. Small molecule inhibitors such as naphthoquinone **1** (shown in red) are valuable tools which can be used to enhance our understanding of this proposed role.

Both biochemical and transgenic evidence suggest a link between NAT and folate (Figure 1). In humans, intracellular folate levels in erythrocytes appear to be inversely correlated with cellular pABA *N-*acetylation activity [26], and folate and its analogues have been identified as inhibitors of *in vitro* arylamine *N-*acetylation measured with several sources of enzymes including pure recombinant (HUMAN)NAT1 [14], [26]. Furthermore, both (HUMAN)NAT1 and (MOUSE)NAT2 are able to acetylate the folate catabolite *para*-aminobenzoyl-1-glutamate (pABAglu) [20] as well as pABA (Figure 1).

Over the last decade or so, the development of mouse models has greatly enhanced our understanding of the biological role of (MOUSE)NAT2 (and, by analogy, (HUMAN)NAT1). Observations in knockout mouse lines have suggested that (HUMAN)NAT1 and its homologue (MOUSE)NAT2 may play a role in folate catabolism [27]. Targeted insertion of a *LacZ* transgene into the (MOUSE)NAT2 locus provided a functional knockout which also acts as a reporter model for expression of the *(MOUSE)Nat2* gene [28]. The resulting NAT2 null mice appear phenotypically normal, but their livers, kidneys and urine contain no detectable *N-*acetyl-pABAglu and they exhibit biochemical abnormalities in 5-methyl tetrahydrofolate and *S*-adenosylmethionine homeostasis [29], [30]. Whole-mount histochemical staining indicates that the transgene is expressed in the closing neural tube of the developing embryo in this line. The closure process occurs normally in the majority of transgenic embryos, but an increased incidence of posterior NTDs and occasional ocular defects is detected when there is a genetic imbalance between the parents [29], [31].

Mice engineered with constructs designed to drive overexpression of (HUMAN)NAT1 exhibit poor survival and the progeny of the few surviving founders only exhibit small increases in hepatic pABA acetylation [32], [33], suggesting that (HUMAN)NAT1 overexpression during development could be harmful, possibly due to disruption of folate homeostasis. Moreover, the overexpression of (HUMAN)NAT1 in mice provokes NTDs usually associated with folate deficiency [32], [34], [35] and has complex effects on the incidence and severity of orofacial clefting [33].

Folate-dependent methylation appears to play a role in the epigenetic regulation of (MOUSE)NAT2 expression: specifically, tissue-specific CpG methylation of the promoter region increases in a strongly cell cycle-associated fashion after functional deletion of the gene or following dietary folate supplements [36], consistent with the aberrant expression of (HUMAN)NAT1 in certain developmental defects and cancers. In humans, promoter hypomethylation associated with increased *(HUMAN)NAT1* mRNA expression has been demonstrated in infiltrating ductal carcinomas of the breast [37].

The overexpression of (HUMAN)NAT1 may be of pathological significance. Proteomic analysis has identified (HUMAN)NAT1 as one of the predominant proteins overexpressed in invasive ductal and lobular breast cancers; moreover, cellular proliferation and the acquisition of etoposide resistance was observed when (HUMAN)NAT1 overexpression was induced in non-cancerous human breast cell lines [38]. In addition, transcriptional profiling has identified (HUMAN)NAT1 as a potential prognostic biomarker in male breast cancer [39].

Studies using human breast tumour-derived cell lines have indicated a 50-fold range of (HUMAN)NAT1 activities among estrogen-receptor (ER)-positive cell lines, the highest activity being observed in the ZR-75-1 line [3], [40]. These observations led various investigators to evaluate small molecule libraries with a view to identifying (HUMAN)NAT1 inhibitors with chemotherapeutic potential [1], [41]–[45]. Particular attention has been paid to the small molecule naphthoquinone **1** because of its striking selectivity for (HUMAN)NAT1/(MOUSE)NAT2 (Figure 1A) and its diagnostic/prognostic potential in ER-positive breast tumours [44], [45].

The aim of the present study was to determine whether (HUMAN)NAT1 and its murine homologue can catalyse the direct hydrolysis of acetyl Coenzyme A in the presence of folate. We characterised enzyme selectivity using a broad panel of eukaryotic and prokaryotic NAT enzymes, and describe here experiments demonstrating that (HUMAN)NAT1 and its homologue (MOUSE)NAT2, but not prokaryotic NATs, can catalyse this reaction even in the absence of any arylamine substrate. We also investigated the catalytic mechanism and final products of the reaction. Folate was not acetylated during the reaction; instead, the acetate released from AcCoA entered the bulk solvent. We propose that folate is a cofactor for the hydrolysis of AcCoA by (HUMAN)NAT1 and its mouse equivalent (MOUSE)NAT2 and suggest this reaction as an endogenous function of this widespread enzyme, differing from that of (HUMAN)NAT2, where the presence of an added arylamine is essential to permit appreciable rates of AcCoA hydrolysis to occur.

## Materials and Methods

### Chemicals

All chemicals were purchased from Sigma-Aldrich unless otherwise stated. Molecular biology reagents were obtained from Promega (Southampton, UK). Competent cells were purchased from Promega and Invitrogen (Carlsbad, USA).

### Expression of Pure Recombinant NAT Enzymes

Recombinant NAT enzymes were prepared as described previously [5], [20], [46]–[53]. Human ((HUMAN)NAT1), murine ((MOUSE)NAT1 and (MOUSE)NAT2) and prokaryotic NATS (from *Salmonella typhimurium*, *Mycobacterium marinum*, *Mycobacterium smegmatis*, *Pseudomonas aeruginosa* and *Mycobacterium tuberculosis* ((SALTY)NAT, (MYCMR)NAT, (MYCSM)NAT, (PSEAE)NAT) and (MYCTU)NAT)) were evaluated.

### Detection of Catalytic AcCoA Hydrolysis by HPLC Analysis

The activity of pure recombinant (HUMAN)NAT1 protein was measured using reverse-phase HPLC as previously described by Grant *et al.* [54]. Enzyme samples were first incubated with folate (500 µM, final 5% DMSO (v/v)) in assay buffer (25 mM Tris-HCl, pH 7.5) at 37°C for 5 min. The reaction was started by adding AcCoA to a final concentration of 400 µM (final volume 100 µL) and allowed to proceed at 37°C for various periods of time (up to 60 min). The reaction was quenched by adding 100 µL of ice-cold aqueous perchlorate (15% w/v), precipitated proteins were removed by centrifugation for 5 min at 12,000 *g* and 20 µL of the supernatant was injected onto a C18 reverse-phase HPLC column.

### Determination of NAT Activity using Colorimetric Assays

Two methods which have previously been demonstrated to give equivalent results [55] were used to determine NAT activity; one quantified the direct disappearance of the arylamine over time, the other estimated the formation of the Coenzyme A (CoA) product. Assays were conducted in triplicate and the rates and kinetic parameters quoted represent the mean of three independent measurements ± standard deviation unless otherwise stated. In some experiments, the ability of *n*-propionyl CoA to replace AcCoA was investigated using the same methods.

#### Determination of NAT activity in terms of substrate acetylation

A modification of the method of Andres *et al.* [56] was used to determine the rates of enzymatic reactions with arylamine substrates. Samples of (MOUSE)NAT2 (5 ng), with pABA as the arylamine substrate (150 µM), were pre-incubated at 37°C in assay buffer (20 mM Tris-HCl, pH 8.0 containing 1 mM DTT) for 5 min in a 96-well plate (Corning). The reaction was initiated by adding pre-warmed AcCoA to a final concentration of 400 µM (final volume 45 µL), allowed to proceed for the appropriate time at 37°C and quenched by adding an equal volume of 20% (w/v) aqueous trichloroacetic acid solution at 0°C. The colorimetric reagent *para*-dimethylaminobenzaldehyde (DMAB) (5% (w/v) in 90% aqueous CH_3_CN solution, 150 µL) was added and after 15 min incubation at room temperature the absorbance was measured at 450 nm in a 96-well plate reader (Tecan Sunrise). The remaining arylamine concentration was calculated by reference to a standard curve. Folate could be detected using a similar method because its pteridine moiety possesses a primary amino group which can react with DMAB to form a coloured product detectable at 450 nm. The reactivity of folic acid with DMAB is lower than that of pABA but sufficient concentration-dependent linearity was observed to permit this reaction to be used to detect free folate and evaluate the potential for folate acetylation.

#### Determination of NAT activity in terms of AcCoA hydrolysis

The formation of CoA was determined spectrophotometrically using the colorimetric agent 5,5′-dithio-bis(2-nitrobenzoic acid) (Ellman’s reagent, DTNB) as described previously [55], with some minor modifications. This method is applicable to a wide range of arylhydrazines and arylhydroxylamines as well as arylamines; for the purposes of this study, the appropriate substrate was chosen according to Westwood *et al.* [57] depending upon the enzyme under investigation. Following preincubation of a reaction mixture containing the relevant enzyme with its arylamine substrate in assay buffer (20 mM Tris-HCl, pH 8.0) for 5 min at 37°C, pre-warmed AcCoA in assay buffer was added to 400 µM final concentration, starting the reaction (final volume 100 µL), which was allowed to proceed at 37°C. After the appropriate incubation time, simultaneous quenching and colour development was achieved by addition of DTNB (5 mM in 100 mM Tris-HCl, 6.4 M guanidine.HCl, pH 7.3; 25 µL). The absorbance was read immediately at 405 nm and the rate of reaction was determined by reference to a standard curve. The same method was used to examine the hydrolysis of *n*-propionyl CoA.

### Use of NMR to Determine Folate-dependent AcCoA Hydrolysis by (MOUSE)NAT2

Prior to the commencement of enzymatic experiments, ^1^ *H*-NMR spectra of all the reagents and potential products of the assay were recorded using a Bruker Avance AVIII 700 spectrometer at the following concentrations: AcCoA (400 µM), folate (150 µM), CoA (400 µM), and acetic acid (AcOH) (400 µM), all in phosphate-buffered saline solution in D_2_O (PBS-D_2_O; pD 7.4) (Figures S1, S2, S3). Each peak was assigned to the corresponding proton consistent with previous full assignments [58]–[60]. Most of the chemical shifts of AcCoA and CoA protons were similar, but the chemical shifts of protons 6″ and 9″ differed and were chosen as markers of these two molecules in a real-time ^1^ *H*-NMR assay. The concentration of folate used in the assay (150 µM) permitted recognition of all spectral bands and the possible AcOH product could be identified from a singlet at 1.97 ppm. The characteristic resonances corresponding to all reagents and products were found to be distinguishable in the spectra of the assay mixture at the concentrations stated; the range from 3.2 to 1.6 ppm was chosen as the most adequate region to follow the enzymatic AcCoA hydrolysis by (MOUSE)NAT2 in the presence of folate (Figure S4).

For the determination of folate-dependent AcCoA hydrolysis activity, the reaction mixture comprised purified recombinant (MOUSE)NAT2 (25 µg), folate (150 µM) and AcCoA (400 µM) in PBS-D_2_O at 37°C. Following the collection of basal data the reaction was initiated by adding AcCoA to a final concentration of 400 µM and monitored by real-time ^1^ *H*-NMR using a Bruker Avance AVII 500 MHz spectrometer. The field was locked by external referencing to the relevant deuteron resonance (D_2_O). The integration values were corrected appropriately and referenced to a common internal standard (DMSO; 5% (v/v)).

### Structural Docking Simulations

All images showing protein structures were generated using PyMOL software [61]. The ground state conformation of folate was predicted using the molecular editor Avogadro. Analysis of the possible interactions between folate and the active site of (HUMAN)NAT1 (pdb: 2PQT) [6] were performed using the licensed software GOLD [62]. A docking site was defined as a region of 10 Å within the active pocket of the enzyme and the generated solutions were ranked by their GOLD Score Fitness functions.

Modelling was also used to compare the shape and electron density of folate with that of CoA. The conformation of CoA used in these studies was that extracted from the co-crystal structure of CoA with (HUMAN)NAT2 [6]. Modelling was carried out using the licensed software FORGE (Cresset Group, Litlington, Cambridgeshire, UK). CoA from its co-crystal structure with (HUMAN)NAT2 was used as the reference molecule, and folate was used as the test ligand. Modelling was carried out comparing the two molecules both in free space and subsequently using (HUMAN)NAT2 as an excluded volume.

## Results and Discussion

### (HUMAN)NAT1 and (MOUSE)NAT2, but not other NATs, can act as Folate-dependent Acetyl CoA Hydrolases

A truncated form of (HUMAN)NAT1 with a shortened *C*-terminus is known to be able to catalyse the hydrolysis of AcCoA without transfer of the acetyl moiety to the arylamine substrate [8]. Such hydrolysis has also been demonstrated with truncated forms of the NAT enzyme from *S. typhimurium* [63], but has not, to date, been observed in routine enzymatic assays using full length mammalian NAT isoforms.

**Figure 2:**
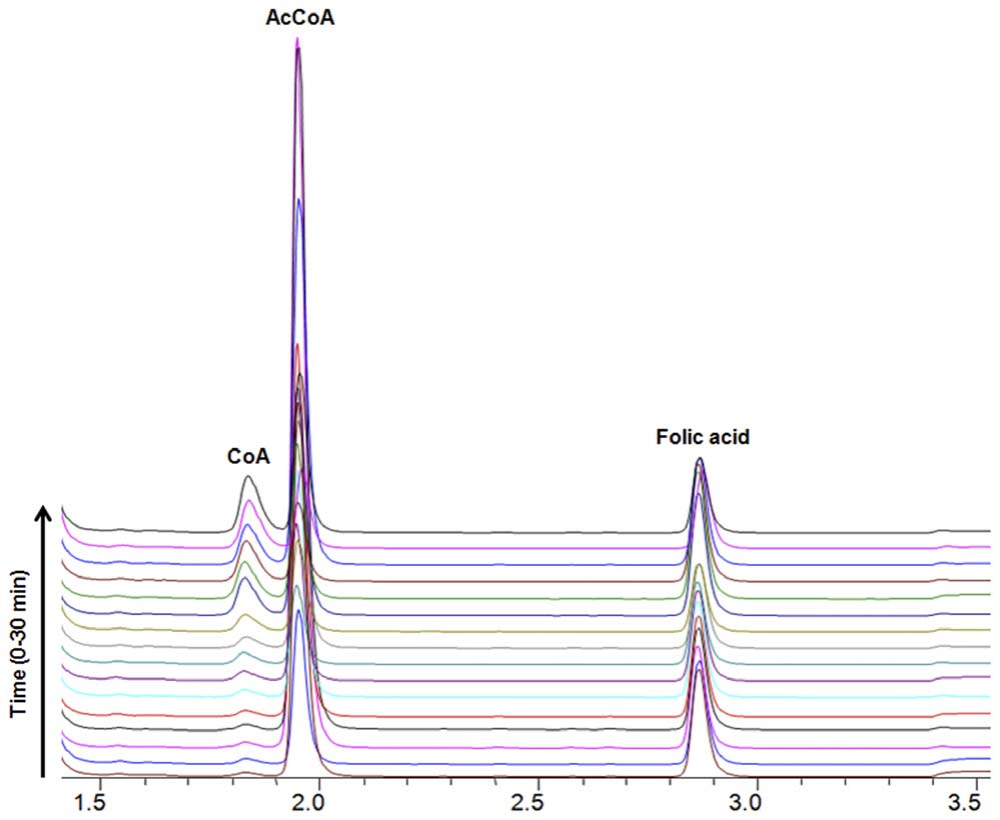
(HUMAN)NAT1 hydrolyses AcCoA in the presence of folate. The time course of AcCoA hydrolysis by (HUMAN)NAT1 in the presence of folate was monitored. The reaction mixture was prepared and incubated as described. Aliquots were taken every 2 minutes over a 30 minute time course and analysed by HPLC.

We initially used HPLC analysis to determine whether pure recombinant (HUMAN)NAT1 could act as an AcCoA hydrolase in the presence of folate alone and found that, over a 30 minute reaction period, the peak corresponding to AcCoA gradually reduced in area while the area of the CoA peak increased (Figure 2). The disappearance of the peak corresponding to AcCoA and appearance of the CoA peak was clear from the sequential HPLC traces; however, no change in the folate peak was observed, indicating that the role of folate in this reaction was not as a substrate. This behaviour was characteristic of (HUMAN)NAT1 as opposed to (HUMAN)NAT2, and was also observed in the presence of other folate analogues or moieties, such as methotrexate, pterin, and pteroic acid, but with lower efficiency (results not shown).

**Figure 3:**
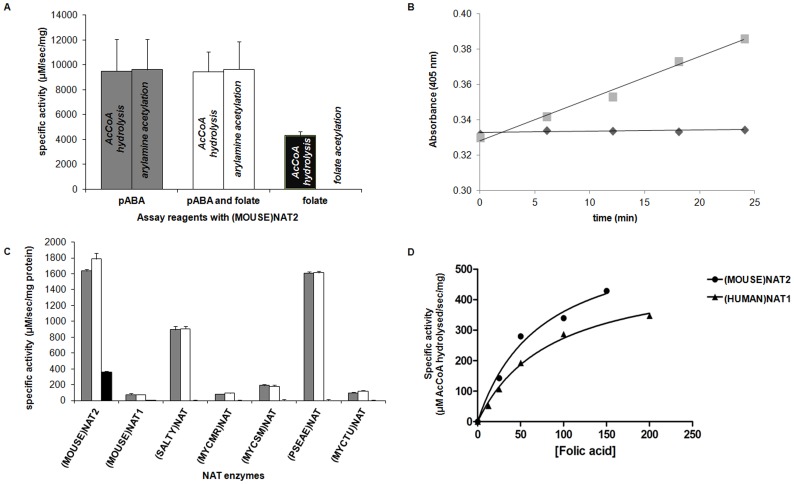
AcCoA hydrolysis by (MOUSE)NAT2 in the presence of folate. (A) Specific pABA (150 µM) acetylation activity by (MOUSE)NAT2 (5 ng) was followed in the absence (grey columns) or in the presence (white columns) of an equimolar concentration of folate. The concentration of AcCoA was 400 µM in all assays. Specific AcCoA (400 µM) hydrolysis activity by (MOUSE)NAT2 (5 ng) was also followed in the presence of folate (150 µM), but in the absence of an arylamine substrate (black columns). For each set of reagents, both primary amine acetylation and AcCoA hydrolysis methods were used in parallel, yielding equivalent results. No acetylation of folate was detected in a corresponding acetylation assay. (B) Time course of assay containing AcCoA (400 µM) and folate (150 µM), with 5 ng (MOUSE)NAT2 (squares); negative control: assay containing AcCoA (400 µM) and folate (150 µM) only, without (MOUSE)NAT2 (diamonds). (C) Activity of mammalian and bacterial NATs as AcCoA hydrolases in the presence of folate. Different NAT enzymes were tested in AcCoA hydrolysis assays in the presence of either the arylamine substrate 5AS (150 µM, grey columns), both 5AS and folate (each at 150 µM, white columns) or folate alone (150 µM, black columns). (D) Initial velocities of AcCoA hydrolysis by (HUMAN)NAT1 (triangles) and (MOUSE)NAT2 (circles) in the presence of different concentrations of folate. A Michaelis-Menten curve was fitted using the formula: y = (V_max_×[folate])/(K_m_+[folate]).

Following the discovery that (HUMAN)NAT1 can hydrolyse AcCoA in the presence of folate, the ability of its homologue, (MOUSE)NAT2, to act as a folate-dependent AcCoA hydrolase was also investigated. Single time point analytical assays were performed with (MOUSE)NAT2 in order to evaluate the possible fate of the reaction participants. In preliminary experiments to determine whether (MOUSE)NAT2 can hydrolyse AcCoA in the presence of folate, reactions were carried out in the presence of: pABA and AcCoA; pABA, folate (in equimolar concentrations) and AcCoA; or folate and AcCoA in the absence of pABA (Figure 3A). The results obtained confirmed the ability of (MOUSE)NAT2 to acetylate the arylamine pABA (150 µM) in the presence of AcCoA and demonstrated that the addition of 150 µM folate to the reaction did not affect this activity, whether measured in terms of substrate acetylation or release of CoA. The third set of conditions demonstrated that (MOUSE)NAT2 could catalyse the time-dependent generation of CoA from AcCoA in the presence of folate but that no acetylation of folate occurred, at least under the single time-point conditions used. These results suggest a possible role of folate as a cofactor for AcCoA hydrolysis by both (HUMAN)NAT1 and (MOUSE)NAT2, rather than an acetylation substrate. The ability of (MOUSE)NAT2, but not (MOUSE)NAT1, to hydrolyse AcCoA in the presence of folate corresponds with the ability of (HUMAN)NAT1 to act as a folate-dependent AcCoA hydrolase, in contrast with (HUMAN)NAT2.

The time course of the AcCoA hydrolysis reaction catalysed by (MOUSE)NAT2 in the presence of folate indicated that CoA release approximated to linearity over 25 minutes (Figure 3B). No hydrolysis of AcCoA was detected in the absence of (MOUSE)NAT2, confirming that hydrolysis of AcCoA in the presence of folate was enzyme-dependent under the conditions used, nor was AcCoA hydrolysis observed with (MOUSE)NAT2 alone, consistent with conventional negative controls used in *N-*acetylation assays with DTNB as a colorimetric quenching agent [55].

The AcCoA hydrolysing capacity of a range of other murine and prokaryotic NATs was evaluated by means of similar enzymatic assays performed in the presence of: 5-aminosalicylic acid (5AS; 150 µM) as the arylamine substrate; both 5AS and folate (each at 150 µM); or folate (150 µM) alone (Figure 3C). No inhibition of 5AS acetylation activity was observed for any NAT isoform in the presence of folate. The only isoform capable of hydrolysing AcCoA in the presence of folate alone without an acceptor arylamine substrate being added to the reaction mixture was (MOUSE)NAT2. The rate of this reaction was estimated to be 360 µM.sec^−1^.mg^−1^. Another acyl donor substrate of (MOUSE)NAT2, *n-*propionyl CoA, was hydrolysed by the enzyme in the presence of folate with a slightly lower activity (290 µM.sec^−1^.mg^−1^) compared to AcCoA.

In order to estimate the folate concentration dependence of AcCoA hydrolysis by (HUMAN)NAT1 and (MOUSE)NAT2, increasing concentrations of folate were tested with each enzyme. The hydrolytic activities measured were consistent with Michaelis-Menten kinetics. Apparent dissociation constants for folate (assumed to be acting as a cofactor) were estimated to be 75 µM for (HUMAN)NAT1 and 71 µM for (MOUSE)NAT2. The corresponding V_max_ values were 583 µM.sec^−1^.mg^−1^ and 613 µM.sec^−1^.mg^−1^, respectively (Figure 3D).

In order to investigate whether the enzyme selectivity of folate-dependent AcCoA hydrolysis is related to specific interactions between the AcCoA substrate and the NAT enzyme, the structures of several CoA-complexed NAT enzymes were compared. This comparison indicated that the binding site for CoA (analogous to AcCoA) involves the *C-*terminus but that the AcCoA binding site differs from one NAT enzyme to another. In addition, the existence of a loop between domains two and three appears to be of great importance with regard to CoA binding specificity [2]: shortening the third domain of (SALTY)NAT, either completely or partially, leads to increased AcCoA hydrolysis and inability to acetylate arylamines following formation of the acetyl-enzyme intermediate [63]. In previous studies, a truncated version of (HUMAN)NAT1 comprising only the first 204 residues was found to hydrolyse AcCoA without concomitant acetylation of the arylamine substrate [8]. Furthermore, Wang *et al*. [64] demonstrated that the acetylated derivative of Syrian hamster NAT Type 2 could be hydrolysed in the presence of arylamines whose weak nucleophilicity delays attack on the acetyl-Cys68 thioester bond. In the absence of an arylamine substrate, hydrolysis of the rapidly formed acetylated intermediate was only detected (at a velocity of 31–67 nM/sec) when high concentrations (4–8 µM) of enzyme were used [65]. Moreover, under these conditions the half-life of the acetylated enzyme intermediate was around 1 min, confirming that the acetyl-enzyme intermediate was stable and suggesting that spontaneous deacetylation was rate-limiting [65], [66]. Enzyme concentrations in the (MOUSE)NAT2 reactions presented in this study were always ≤3 nM and negative control assays confirmed that the production of free CoA by (MOUSE)NAT2 as a side product of the reaction without the need of folate was close to or below the limit of detection.

It is interesting that the exclusive function of both (HUMAN)NAT1 and (MOUSE)NAT2, which have identical *C*-termini, with respect to AcCoA hydrolysis in the presence of folate corresponds with the ability of these, but not other NAT isoforms, to acetylate the bulkier arylamine pABAglu [20]. In the human isoforms, this is thought to be a consequence of the unique presence of three key residues which have bulky side chains (Phe125, Arg127 and Tyr129) in the active site of (HUMAN)NAT1 compared with that of (HUMAN)NAT2, which has serines at these positions [6], making the substrate binding pocket of (HUMAN)NAT1 smaller than that of (HUMAN)NAT2 and conferring this substrate selectivity on (HUMAN)NAT1 compared with (HUMAN)NAT2.

### Fate of the Acetyl Group Removed during NAT-mediated AcCoA Hydrolysis

**Figure 4:**
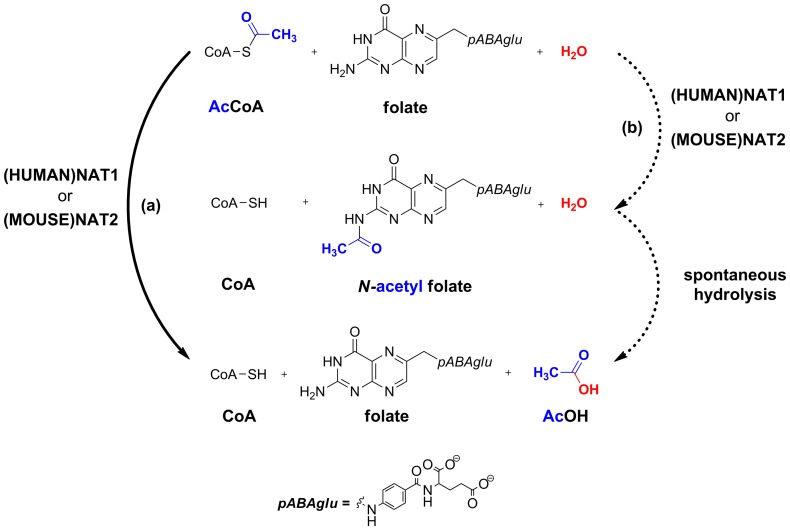
Two hypotheses for the mechanism of the AcCoA hydrolysis reaction catalysed by (MOUSE)NAT2 in the presence of folate. Pathway (a) corresponds to hydrolysis of AcCoA with direct release of AcOH into the bulk solvent; Pathway (b) corresponds to the formation of an unstable *N-*acetylfolate intermediate which immediately decomposes, releasing free AcOH.

The finding that (HUMAN)NAT1 and (MOUSE)NAT2 could hydrolyse AcCoA in the presence of folate but without an arylamine substrate raised the question of whether the acetyl group is released directly into the bulk solvent (Figure 4, Pathway (a)) or forms an unstable *N-*acetylfolate intermediate which immediately decomposes, releasing free AcOH (Figure 4, Pathway (b)).

**Figure 5:**
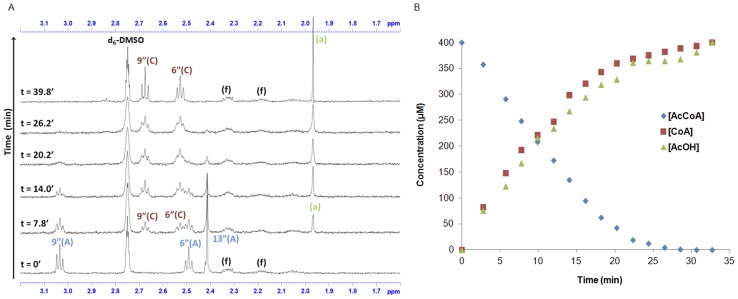
Real-time *^1^H*-NMR analysis of (MOUSE)NAT2 activity as a folate-dependent AcCoA hydrolase performed in PBS-D_2_O. (A) Purified recombinant (MOUSE)NAT2 (25 µg) was mixed with folate (150 µM) and AcCoA (400 µM) in buffer PBS-D_2_O (pD 7.4) and incubated at 37°C. NMR data were collected before adding the enzyme (t = 0 min), and subsequently 5 min after enzyme addition. A selected region of the spectra (3.2 to 1.6 ppm) is shown, the signals being labelled as follows: folate (f), AcCoA (A), CoA (C), AcOH (a). (B) Enzymatic reaction progress curves of folate-dependent AcCoA hydrolytic activity by (MOUSE)NAT2. Folate-dependent AcCoA hydrolysis by (MOUSE)NAT2 was monitored by following the characteristic peaks of both reagent and products *versus d_6_*-DMSO as an internal standard with appropriate correction. Diamonds: AcCoA; Squares: CoA; Triangles: AcOH.

Preliminary HPLC studies did not reveal any *N-*acetylated folate species after AcCoA hydrolysis by (HUMAN)NAT1, nor was AcOH detectable using this analytical technique; however, the biochemical techniques used with (MOUSE)NAT2 were also unable to provide information about the intermediate steps of the catalytic mechanism. In order to resolve this issue, ^1^ *H*-NMR analysis was performed; (MOUSE)NAT2 was used in this experiment because sufficiently large amounts of pure recombinant protein (25 µg) were available to permit ^1^ *H*-NMR analysis. This allowed chemical modifications occurring during the catalytic reaction to be analysed in real time, revealing that the disappearance of acetyl C*H*_3_ from AcCoA (at 2.41 ppm) occurred concomitantly with the appearance of a C*H*_3_ peak (at 1.97 ppm) corresponding to that of free AcOH (Figure 5A). This was consistent with the hypothesis that (MOUSE)NAT2 could catalyse direct hydrolysis of AcCoA in the presence of folate, and that the acetyl group of AcCoA was transferred to the bulk solvent as AcOH. In contrast, no change in the chemical shifts of folate protons was observed, suggesting that the chemical structure of folate was not modified during the reaction; formation of an *N-*acetylated intermediate of folate was therefore considered unlikely. However, the formation of a transient *N-*acetylfolate intermediate could not be excluded at this stage because of the possibility that it could be hydrolysed in D_2_O too rapidly to be detected on the NMR timescale.

In order to visualise the kinetics of folate-dependent AcCoA hydrolysis by (MOUSE)NAT2, reaction progress curves were drawn using the integration values of characteristic resonances arising from AcCoA, CoA and AcOH over time in relation to those of DMSO (5% (v/v)) as an internal standard. The peaks corresponding to the acetyl C*H*_3_ groups of AcCoA and AcOH, and the methylene groups in the α position relative to sulfur in both AcCoA and CoA (numbered as 6″ and 9″ in Figures S2–S3), were also taken into account. The resulting curves indicated non-linear progression of the reaction, both in terms of loss of starting reagent and formation of products, consistent with Michaelis-Menten kinetics. The disappearance of the initial AcCoA was apparently complete after ∼23 min and the formation of both products reached a maximum after the same period of time. When all the integrations were converted into concentration values and plotted against time (Figure 5B), the initial reaction velocity was calculated to be 21.6±2.1 µM.min^−1^.

One possibility is that the primary amine moiety of folate is initially acetylated to form an *N-*acetylfolate intermediate which is subsequently hydrolysed; however, we did not detect such an intermediate by any method used, including *^1^H*-NMR. This may be because no such intermediate exists; alternatively, if it does, it may be turned over too rapidly for detection on the NMR timescale and/or may never accumulate to a high enough level to be detected by *^1^H*-NMR. Given that the time for running a *^1^H*-NMR spectrum is around 4 sec, the initial rate of AcCoA hydrolysis by (MOUSE)NAT2 suggests an estimate of ∼1.4 µM for the concentration of the possible *N-*acetylfolate intermediate, corresponding to ∼1% of the initial amount of folate. This lies at the very limit of detection by *^1^H*-NMR and is certainly beyond the limit of quantification by this method using standard techniques [67]. These data therefore confirm that CoA and AcOH were produced during (MOUSE)NAT2-mediated catalysis in the presence of folate; however, it was not possible to determine whether the reaction proceeded *via* direct hydrolysis of the AcCoA with release of the acetyl group as free AcOH to the bulk solvent or whether a transient intermediate was formed. Since the primary amine of folic acid would be predicted to be less nucleophilic than the primary amine of pABA, due to enhanced resonance stabilisation of the nitrogen lone pair by the pterdine moiety, folate is less likely to act as a nucleophile in the hydrolysis of acetyl-Cys68 than pABA. Taking all this evidence together, therefore, we consider that (MOUSE)NAT2, and by analogy (HUMAN)NAT1, are likely to act as AcCoA hydrolases in the presence of folate without forming an *N-*acetylated folate intermediate.

### Inhibition of AcCoA Hydrolysis by a Selective Naphthoquinone Inhibitor of (HUMAN)NAT1 and (MOUSE)NAT2

**Figure 6:**
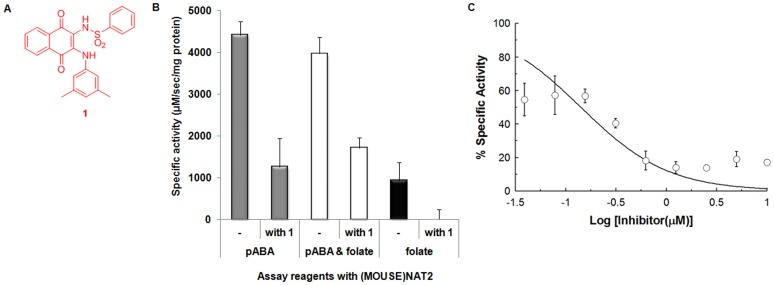
Inhibition of (MOUSE)NAT2-dependent AcCoA hydrolysis by the small molecule inhibitor naphthoquinone 1. (A) Structure of naphthoquinone **1**, a selective inhibitor of (HUMAN)NAT1. (B) Screening of different enzymatic assays with (MOUSE)NAT2 in the presence of naphthoquinone **1**. AcCoA hydrolysis assays were carried out in order to monitor the inhibitory potency of naphthoquinone **1** at 10 µM. AcCoA was present at 400 µM in all assays. Specific pABA (150 µM) acetylation activities by (MOUSE)NAT2 (5 ng) were performed in the absence (grey columns) or presence (white columns) of an equimolar concentration of folate. Specific AcCoA hydrolysis activity was also detected in the presence of folate (150 µM) in a DTNB assay without an arylamine substrate (black columns). The percentage inhibition was calculated in terms of the reduction in (MOUSE)NAT2 specific activity measured against a control in which DMSO (5% (v/v)) alone was added to the assay. (C) Naphthoquinone **1** as an inhibitor of the folate-dependent AcCoA hydrolytic activity of (MOUSE)NAT2. AcCoA hydrolysis assays were performed using (MOUSE)NAT2 (510 ng) and AcCoA (400 µM) in the presence of folate (150 µM) and serial dilutions of naphthoquinone **1**. Data points represent the mean ± standard deviation of triplicate assays. Percentage inhibition was determined as the ratio of specific activity with the inhibitor related to specific activity without inhibitor (100%); 100% activity was 198 µM.sec^−1^.mg^−1^. The regression equation used was: y = 100/[1+10∧(x-log(IC_50_)].

In order to characterise the folate-dependent hydrolysis of AcCoA further, we made use of a small molecule inhibitor (for background information on small molecule inhibitors of NATs, see [2]). Naphthoquinone **1** (Figure 6A) is a selective competitive inhibitor of *N-*acetylation by (HUMAN)NAT1 and (MOUSE)NAT2 [44], and Scatchard analysis indicates that it binds to a single site, specifically the active site, on (MOUSE)NAT2. In addition, **1** shows the remarkable quality of changing colour from red to blue on binding specifically to (HUMAN)NAT1 or (MOUSE)NAT2, whereas it does not change colour on binding to other isoforms, in particular (HUMAN)NAT2 [45].

The availability of large amounts of stable pure recombinant (MOUSE)NAT2 allowed us to investigate the ability of naphthoquinone **1** to inhibit (MOUSE)NAT2-dependent AcCoA hydrolysis in three assay formats: arylamine *N-*acetylation in the absence of folate, arylamine *N-*acetylation in the presence of folate, and the folate-dependent AcCoA hydrolysis reaction (Figure 6B). As demonstrated previously, naphthoquinone **1** is a potent inhibitor of (MOUSE)NAT2 in the arylamine *N-*acetylation reaction; however, the extent of inhibition was reduced by at least 20% in the presence of added folate. In contrast, folate-dependent AcCoA hydrolysis by (MOUSE)NAT2 was totally inhibited by naphthoquinone **1**.

In order to verify that inhibition of folate-dependent AcCoA hydrolysis by naphthoquinone **1** was not an artefact of the enzymatic assay, its concentration dependence was characterised (Figure 6C). This analysis indicated an IC_50_ of 0.14 µM (p<0.01). This was roughly seven-fold lower than the IC_50_ of naphthoquinone **1** for arylamine *N-*acetylation (0.99 µM) [44].

### In silico Docking of Folate within the Active Site of (HUMAN)NAT1

The mechanism of AcCoA hydrolytic catalysis depends both on the binding site and the sequence in which AcCoA and folate bind to the enzyme. Folate-dependent AcCoA hydrolysis corresponds to the first step of the conventional ping-pong bi-bi mechanism of NAT catalysis in the presence of an arylamine substrate [2]. However, in an arylamine *N-*acetylation reaction the aromatic primary amine is subsequently believed to attack the thioester functional group of the acetyl-Cys68 intermediate, resulting in transfer of the *N-*acetyl group to the aromatic amine [64], whereas the proposed second step in an AcCoA hydrolysis reaction by (MOUSE)NAT2 in the presence of folate is deacetylation of the acetyl-Cys68 by a molecule of water to form AcOH.

This hypothetical mechanism requires a water molecule to gain access to the active site acetyl-Cys68 in order to release the acetyl group and contrasts with the situation pertaining in the case of the arylamine *N-*acetylation reaction in which, following the acetylation of the catalytic cysteine, only highly hydrophobic molecules such as arylamines and arylhydrazines can access the active site pouch [6], [57], [68].

The observation that no appreciable AcCoA hydrolysis occurs in control reactions with (MOUSE)NAT2 in the absence of an arylamine substrate (or folate) is consistent with this hypothesis [55]. Conversely, the observed hydrolysis of acetyl-Cys68 in the presence of folate, despite the hydrophobicity of the active site pocket, suggests that folate may modify the hydrophobicity of the acetylated enzyme’s active site, thereby allowing water to enter the active site pocket and cleave acetyl-Cys68, possibly as a consequence of altered folding induced by the binding of folate.

**Figure 7:**
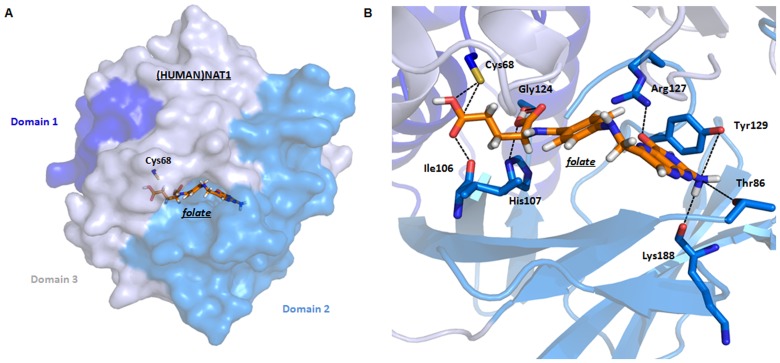
*In silico* docking of folate into the active site of (HUMAN)NAT1. (A) Folic acid was docked into the active site of (HUMAN)NAT1 using the program GOLD [62]; the highest scoring solution is shown. The structure of (HUMAN)NAT1 (pdb code: 2PQT) [6] is shown in surface format with the three domains labelled and coloured in different shades of blue. The domains are numbered from the amino terminus with Domain 3 corresponding to the *C-*terminus of the protein. Folic acid is labelled with carbon atoms in orange, nitrogen in blue, oxygen in red, and hydrogen in white. (B) Maximised view of the (HUMAN)NAT1 active site with folic acid docked. The structure of (HUMAN)NAT1 is shown in cartoon format with the three domains coloured as above. The side chains of the key residues involved in predicted folic acid binding within the catalytic pocket are labelled with nitrogen in blue, oxygen in red, and sulfur in yellow. Folic acid is labelled with carbon atoms in orange, nitrogen in blue, oxygen in red, and hydrogen in white.

We undertook modelling studies in an effort to predict the nature of the interactions between (HUMAN)NAT1 and folate in the case that it binds to the catalytic active site, which is likely because the known (HUMAN)NAT1 specific substrates, pABAglu and pABA, are chemical moieties of folate (Figure 1A). Modelling using GOLD software suggests that the γ-COOH of folate could form hydrogen bonds with the backbone carbonyl of Ile106 and the thiolate moiety of Cys68, and the α-COOH of folate could interact with the backbone carbonyl of Gly124 and the imidazole side chain of His107. Additionally, the pteridine carbonyl of folate could potentially interact with the guanidine moiety of Arg127 and the primary amine of folate with the backbone carbonyl of Lys188 and the hydroxyl groups of Thr96 and Tyr129 (Figure 7).

**Figure 8:**
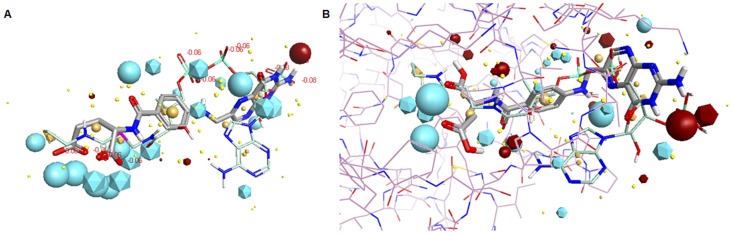
Structural similarity between folate and AcCoA. (A) Predicted alignment between folic acid and CoA in free space using the software program FORGE, based on the conformation of CoA as it is bound in the co-crystal structure with (HUMAN)NAT2 (pdb code: 2PFR). Folic acid is shown in thick sticks, with carbons in grey, and CoA is shown in thin sticks, with carbon atoms in green. Nitrogen atoms are shown in blue, oxygens in red, and hydrogens in white. Regions of negative charge are shown in cyan, regions of positive charge are shown in dark red, and lipophilic regions are shown in yellow. Icosahedra represent the electronic fields of CoA and spheres represent those of folic acid. (B) Predicted alignment between folic acid and CoA using (HUMAN)NAT2 as an excluded volume. Folic acid, CoA and their respective electron density distributions are represented as above. The (HUMAN)NAT2 structure is represented in thin sticks with the carbon atoms in lilac.

Comparing the conformation of CoA as it is bound to the active site of (HUMAN)NAT2, using an available co-crystal structure (pdb code:2PFR), against free folate suggests marked similarities between the shape and electron density of the two species (Figure 8A). Repeatable simulations show that regions of positive charge, negative charge and neutral regions for the two ligands align well in space. Furthermore, when (HUMAN)NAT2 was used as an excluded volume in this modelling, the same conclusions could also be reliably drawn (Figure 8B).

It should be noted that these FORGE experiments involved a comparison between the conformation of CoA as bound to (HUMAN)NAT2 and that of free folate; thus the simulations pertain to free folate, not the predicted conformation of hypothetical enzyme-bound conformations of folate. We used (HUMAN)NAT2 as an excluded volume to ensure that folate and CoA were excluded from the same space, permitting a reliable comparison between the shapes of the two molecules. This does not necessarily suggest that folate is binding to the active site of (HUMAN)NAT2 in this way; interestingly, when folate is docked into the active site of (HUMAN)NAT2 it does give a good predicted fit, but this does not necessarily imply binding of folate to (HUMAN)NAT2 and even if folate does bind to (HUMAN)NAT2, it may not induce the conformational changes required for AcCoA hydrolysis.

It is not possible to state whether our prediction regarding the orientation of folate within the active site of (HUMAN)NAT1 is an accurate representation of the real-life situation since no structures of (HUMAN)NAT1 or other NAT enzymes complexed with folate are yet available. Docking studies based on the structure of an acetylated form of (HUMAN)NAT1 would be particularly informative in this context. Biochemical studies have yielded discordant results concerning the mode of inhibition of eukaryotic NATs by folate; however, the data presented in this paper suggest a possible explanation in that folate-dependent inhibition of arylamine *N-*acetylation may be due to concomitant depletion of AcCoA *via* hydrolysis. In particular, some previous folate inhibitor studies with human NATs [26], which measured NAT activity in terms of arylamine *N-*acetylation, may not have taken into account the possibility of direct hydrolysis of AcCoA.

A representative crystal structure of a folate-bound NAT enzyme is required for a complete definition of the folate binding site of (HUMAN)NAT1, which is needed before a hypothesis for the catalytic mechanism for AcCoA hydrolysis by (HUMAN)NAT1 can be proposed. Although folate has a much higher molecular weight (441.4 g.mol^−1^) than selective (HUMAN)NAT1 arylamine substrates (<266.1 g.mol^−1^ (pABAglu)) [6], our docking simulations have highlighted potentially favourable complementary binding partners for the polar functional groups of folate in the hydrophobic catalytic pocket of (HUMAN)NAT1 *via* hydrogen bond interactions. It is possible that the accommodation of a large molecule such as folate could induce further opening of the (HUMAN)NAT1 active site and mediate the access of a molecule of water to the cavity. Furthermore, modelling studies suggest that the electron density distribution of folate may resemble that of CoA, allowing it to bind to the active sites of human NATs in a similar manner.

Crystallographic structural data are therefore required to clarify the mechanism by which folate selectively facilitates AcCoA hydrolysis by (HUMAN)NAT1. More broadly, our data raise questions about the possible role that certain NAT enzymes could play at the interface between AcCoA homeostasis and folate metabolism.

Folic acid has been reported to act as a free radical scavenger [69], [70], and it is interesting to hypothesise a link between the discovery that (HUMAN)NAT1 and (MOUSE)NAT2 can act as AcCoA hydrolases in the presence of an appropriate amount of folic acid and a potential role for (HUMAN)NAT1/(MOUSE)NAT2 and folate in the regulation of AcCoA/CoA homeostasis in cells, especially under conditions of oxidative stress. The availability of naphthoquinone **1** as a selective inhibitor of folate-dependent AcCoA hydrolysis by (HUMAN)NAT1/(MOUSE)NAT2 adds to the available tool kit for the further investigation of such possibilities. Indeed, (HUMAN)NAT1 has been postulated as a novel biomarker in ER-positive breast cancer [40] and the development of analogues of the naphthoquinone inhibitor **1** for potential diagnostic and chemotherapeutic applications in breast cancer treatment is a current area of interest [71].

## Conclusions

The experiments described in this paper demonstrate that (HUMAN)NAT1 and its homologue (MOUSE)NAT2 can catalyse the direct hydrolysis of acetyl Coenzyme A in the presence of folate. This folate-dependent activity is exclusive to these two isoforms; no AcCoA hydrolysis was found when (HUMAN)NAT2, (MOUSE)NAT1 or recombinant bacterial NATs were incubated with folate. Proton NMR allowed chemical modifications occurring during the catalytic reaction to be analysed in real time; the disappearance of acetyl C*H*_3_ from AcCoA occurred concomitantly with the appearance of a C*H*_3_ peak corresponding to that of free AcOH, suggesting that folate is not acetylated during the reaction. We propose that folate is a cofactor for this reaction and suggest it as an endogenous function of this widespread enzyme. Furthermore, *in silico* docking of folate within the active site of (HUMAN)NAT1 suggests that folate may bind at the enzyme’s active site and facilitate AcCoA hydrolysis.

The evidence presented in this paper both adds to our growing understanding of the endogenous roles of (HUMAN)NAT1 and (MOUSE)NAT2 and expands the catalytic repertoire of these enzymes, demonstrating that they are by no means just xenobiotic metabolising enzymes but probably play an important role in cellular metabolism. Our data highlight the important question of whether, and how, the NAT enzymes play a role at the interface between AcCoA homeostasis and folate metabolism [2]. Furthermore, the characterisation of naphthoquinone **1** as an inhibitor of folate-dependent AcCoA hydrolysis reinforces the value of this compound as a powerful chemical tool which is likely to open up a range of future avenues of exploration *via* selective modulation of (HUMAN)NAT1 and (MOUSE)NAT2 function, both for improved understanding of the developmental role of these enzymes and for better chemotherapeutic approaches to ER-positive breast cancer.

## Supporting Information

Figure S1 **Structure and proton NMR spectra of folic acid.** 500 MHz *^1^H*-NMR spectrum of 400 µM AcCoA in D_2_O, pD 7.4, 25°C (room temperature). Fully resolved spectral assignments were made on the basis of previous studies [72], [73].
(TIF)

Figure S2 **Structure and proton NMR spectra of AcCoA.** 500 MHz *^1^H*-NMR spectrum of 400 µM AcCoA in D_2_O, pD 7.4, 25°C (room temperature). Fully resolved spectral assignments were made on the basis of previous studies [58], [59]. The spectral bands of proton 2′, 3′ and 4′ were assumed to be hidden by the D_2_O signal. Traces of Tris.HCl buffer from AcCoA stock solutions were observed.
(TIF)

Figure S3 **Structure and proton NMR spectra of CoA.** 700 MHz spectrum of 400 µM CoA in D_2_O, pD 7.4, 25°C (room temperature). Fully resolved spectral assignments were made on the basis of previous studies [58]–[60].
(TIF)

Figure S4 **NMR spectra of reagents and possible products present in an AcCoA hydrolysis assay with (MOUSE)NAT2.** NMR spectra were recorded with a Bruker AVC 500 spectrometer at 500 MHz or, for CoA, an AV700 spectrometer at 700 MHz at the optimal concentrations used in the AcCoA enzymatic assay: 400 µM for AcOH, CoA, AcCoA, and 150 µM for folate. The blue square highlights the range of chemical shifts chosen to follow the enzymatic assay over time.
(TIF)
