# Supplementary figures and images for: From Arylamine N-Acetyltransferase to Folate-Dependent Acetyl CoA Hydrolase: Impact of Folic Acid on the Activity of (HUMAN)NAT1 and Its Homologue (MOUSE)NAT2
Source: PLoS One. 2014 May 13;9(5):e96370. doi: 10.1371/journal.pone.0096370 (PMC4019507; doi:10.1371/journal.pone.0096370)

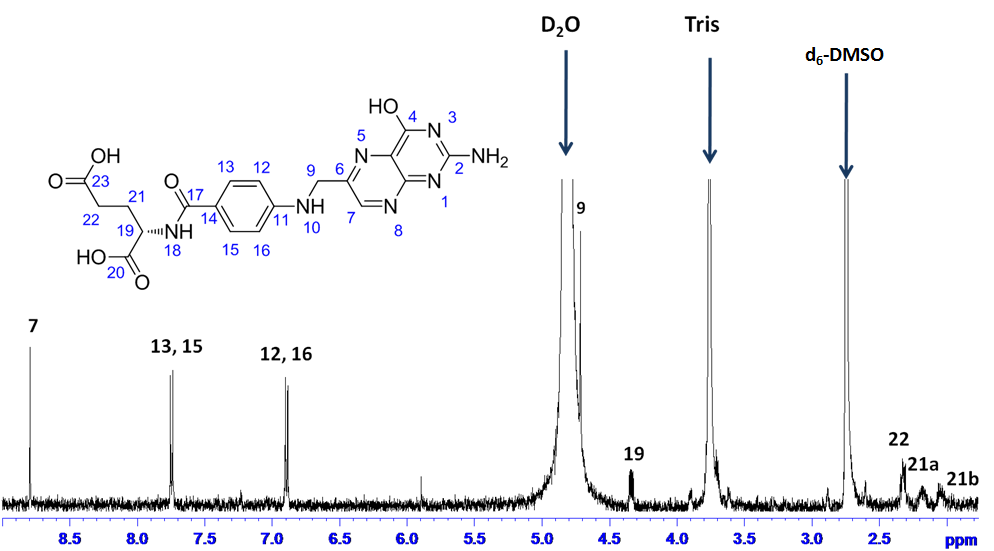

Supplement: Figure S1 — Structure and proton NMR spectra of folic acid. 500 MHz 1H-NMR spectrum of 400 µM AcCoA in D2O, pD 7.4, 25°C (room temperature). Fully resolved spectral assignments were made on the basis of previous studies [72], [73]. (TIF) [file pone.0096370.s001.tif]

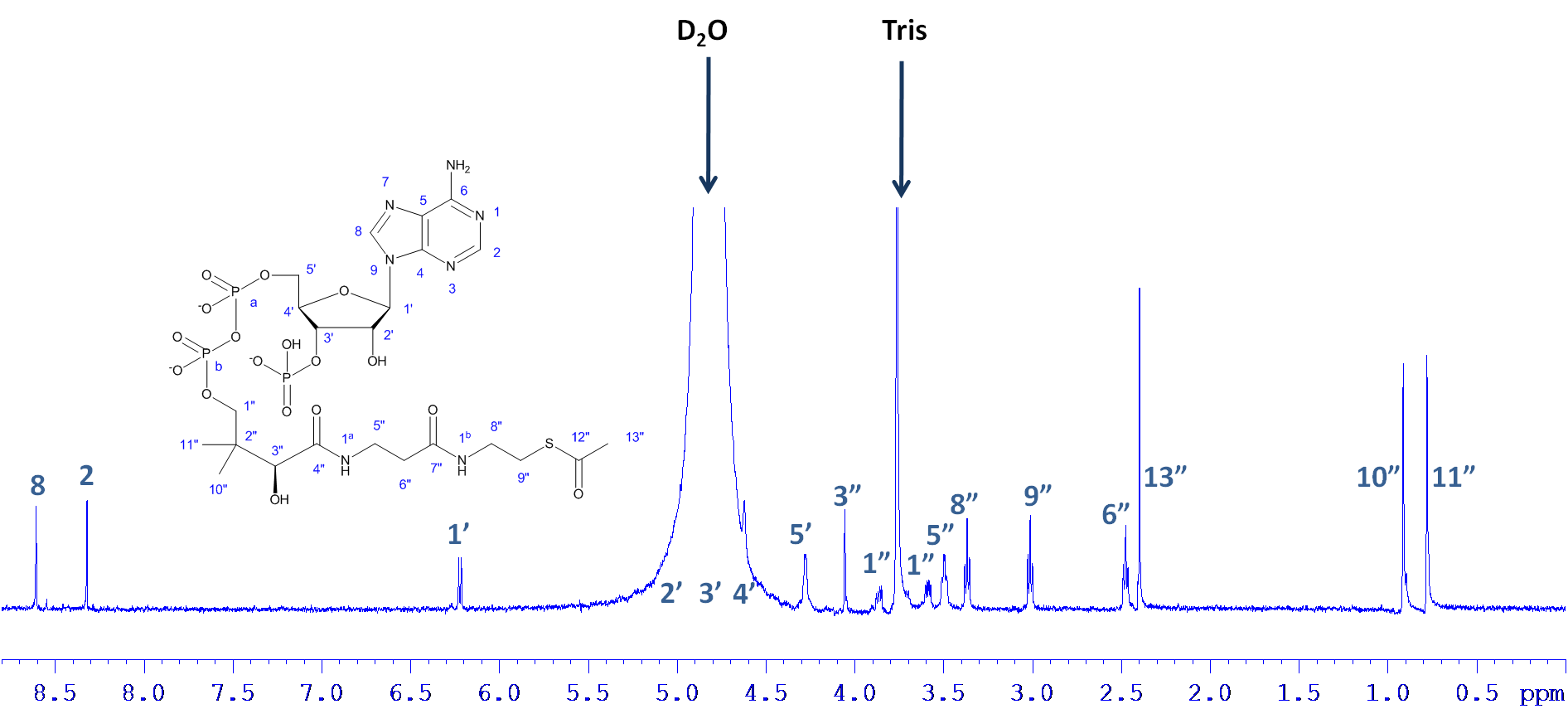

Supplement: Figure S2 — Structure and proton NMR spectra of AcCoA. 500 MHz 1H-NMR spectrum of 400 µM AcCoA in D2O, pD 7.4, 25°C (room temperature). Fully resolved spectral assignments were made on the basis of previous studies [58], [59]. The spectral bands of proton 2′, 3′ and 4′ were assumed to be hidden by the D2O signal. Traces of Tris.HCl buffer from AcCoA stock solutions were observed. (TIF) [file pone.0096370.s002.tif]

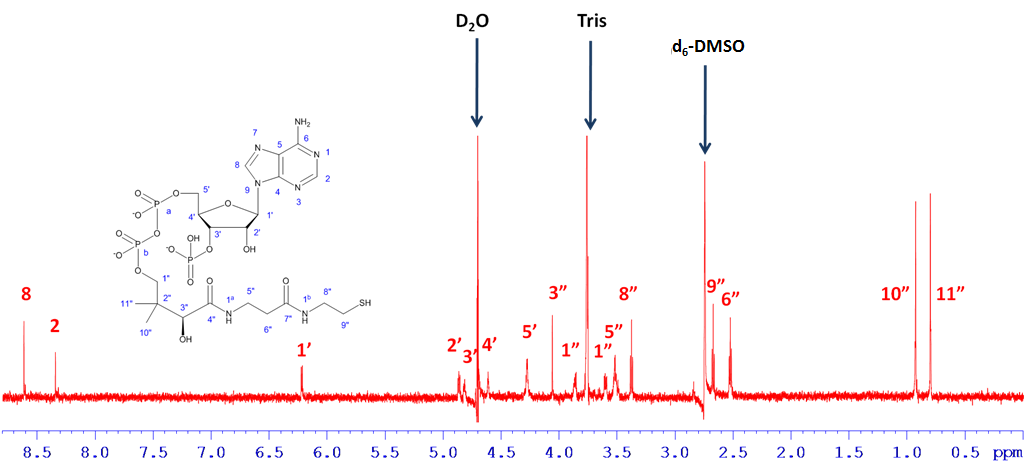

Supplement: Figure S3 — Structure and proton NMR spectra of CoA. 700 MHz spectrum of 400 µM CoA in D2O, pD 7.4, 25°C (room temperature). Fully resolved spectral assignments were made on the basis of previous studies [58]–[60]. (TIF) [file pone.0096370.s003.tif]

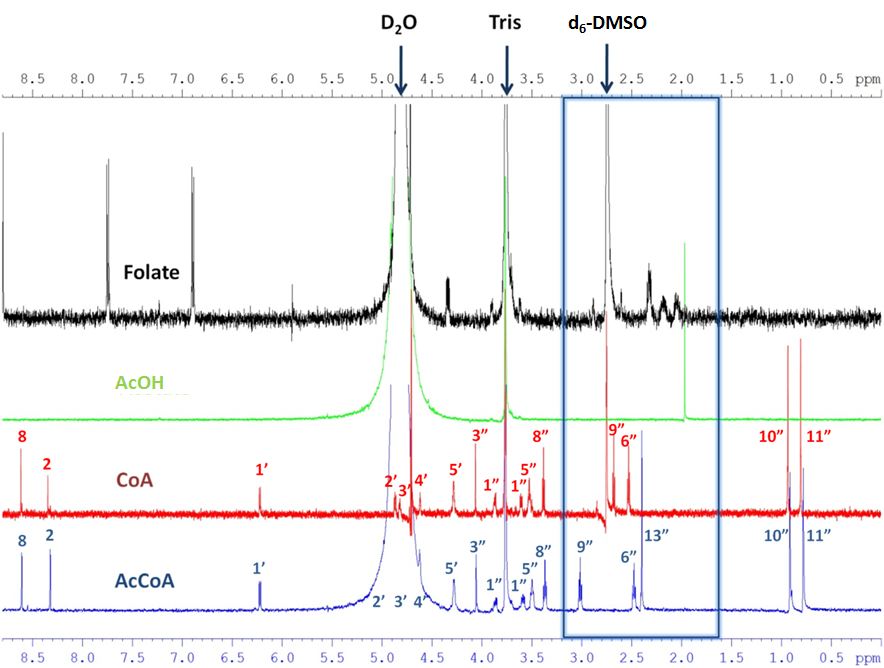

Supplement: Figure S4 — NMR spectra of reagents and possible products present in an AcCoA hydrolysis assay with (MOUSE)NAT2. NMR spectra were recorded with a Bruker AVC 500 spectrometer at 500 MHz or, for CoA, an AV700 spectrometer at 700 MHz at the optimal concentrations used in the AcCoA enzymatic assay: 400 µM for AcOH, CoA, AcCoA, and 150 µM for folate. The blue square highlights the range of chemical shifts chosen to follow the enzymatic assay over time. (TIF) [file pone.0096370.s004.tif]
